# The Enzyme Lysine Malonylation of Calvin Cycle and Gluconeogenesis Regulated Glycometabolism in *Nostoc flagelliforme* to Adapt to Drought Stress

**DOI:** 10.3390/ijms24098446

**Published:** 2023-05-08

**Authors:** Meng Wang, Qiang Zhu, Ning Yao, Wangli Liang, Xiaoxia Ma, Jingjing Li, Xiaoxu Li, Lingxia Wang, Wenyu Liang

**Affiliations:** School of Life Sciences, Ningxia University, Yinchuan 750021, China

**Keywords:** terrestrial cyanobacteria, lysine malonylation, drought stress, carbon metabolism, photosynthesis, molecular function

## Abstract

Lysine malonylation (Kmal) is an evolutionarily conserved post-translational modification (PTM) that has been demonstrated to be involved in cellular and organismal metabolism. However, the role that Kmal plays in response to drought stress of the terrestrial cyanobacteria *N. flagelliforme* is still unknown. In this study, we performed the first proteomic analysis of Kmal in *N. flagelliforme* under different drought stresses using LC-MS/MS. In total, 421 malonylated lysine residues were found in 236 different proteins. GO and KEGG enrichment analysis indicated that these malonylated proteins were highly enriched in several metabolic pathways, including carbon metabolism and photosynthesis. Decreased malonylation levels were found to hinder the reception and transmission of light energy and CO_2_ fixation, which led to a decrease in photosynthetic activity. Kmal was also shown to inhibit the flux of the TCA cycle and activate the gluconeogenesis pathway in response to drought stress. Furthermore, malonylated antioxidant enzymes and antioxidants were synergistically involved in reactive oxygen species (ROS) scavenging. Malonylation was involved in lipid degradation and amino acid biosynthesis as part of drought stress adaptation. This work represents the first comprehensive investigation of the role of malonylation in dehydrated *N. flagelliforme*, providing an important resource for understanding the drought tolerance mechanism of this organism.

## 1. Introduction

As global climate change intensifies, the frequency and intensity of severe water shortages caused by extreme weather are expected to increase, which will exert greater pressure on the growth and yield of sessile plants [1,2]. In plants, water shortage stress results in the inhibition of photosynthesis and metabolism, the production of excessive reactive oxygen species (ROS), and increased oxidative damage to proteins, lipids, nucleic acids, and other cell macromolecules. These changes accumulate, eventually leading to a reduction in overall plant health and growth [3,4]. To combat this, plants have developed a variety of strategies to adapt to abiotic stress at the physiological and molecular levels [5,6]. Drought response involves complex regulatory networks, in which post-translational modification (PTM) enables plants to respond quickly to changing environmental conditions [7]. PTM has repeatedly been demonstrated to be critical in plant adaptation to a variety of environmental stresses [8].

Lysine malonylation is a recently discovered PTM in which a malonyl group is added to lysine, resulting in a negatively charged malonylated lysine with drastic structural changes. These changes can have significant impacts on the function of the substrate protein, including localization, enzyme activity, protein stability, and many other biochemical properties [9,10,11]. Recently, there has been increased interest in understanding the regulatory role that Kmal plays in photosynthetic organisms, such as *Synechocystis* sp. 6803 [12], maize [13], common wheat [11], and rice [14]. These malonylome studies have shown that Kmal exists on proteins with a wide variety of functions. Additionally, it is a highly conserved PTM that impacts many biological processes, including carbon metabolism, fatty acid metabolism, the Calvin cycle, and photosynthesis [12,14,15]. Previous studies reported that malonyl modification could affect enzymatic activity in Mouse [16] and *E. coli* [17], and impact protein synthesis in *S. aureus* [10]. Ma et al. [12] mutated the conserved lysine malonylated site K205 of phosphoglycerate kinase (PGK), and found that the K205E mutant had dramatically decreased enzymatic activity, resulting in altered cellular metabolism. In addition, site-specific mutation in *S. erythraea* showed that malonylation negatively regulated the enzymatic activities of the acetyl-CoA synthetase (Acs) and glutamine synthetase (Gs) [15]. Taken together, these findings clearly demonstrate that Kmal plays critical regulatory roles in both prokaryotes and eukaryotes.

*Nostoc flagelliforme* is a desiccation-tolerant filamentous terrestrial cyanobacteria that is distributed in arid and semi-arid desert steppes of the west and northwest parts of China [18]. It has been consumed by humans for over 2000 years, and its potential medicinal properties were first recognized over 400 years ago [19]. It participates in the formation of desert biological soil crusts and has important economic and ecological value [20]. In recent years, this species has been employed as an ideal material for deciphering stress adaptation in dryland environments [18,21]. In order to adapt to harsh arid environments, *N. flagelliforme* closely regulates its metabolism and protein functions at the post-translational level. A previous systematic analysis of lysine phosphorylated proteins of *N. flagelliforme* under different drought stresses showed that these phosphoproteins are involved in a variety of dehydration-induced signaling processes, such as photosynthesis, sucrose metabolism, and the ROS scavenging system [22]. Furthermore, Wang et al. [23] found that differentially accumulated acetylproteins (DAAPs) in colonies were significantly enriched in carbon metabolism and ROS scavenging systems. Changes in succinylated proteins in response to dehydration indicate that *N. flagelliforme* adapts to dehydration by increasing glucose accumulation and pentose phosphate pathway flux, while decreasing photosynthetic rate [24]. Although these modification studies have improved our understanding of the molecular mechanisms related to the water stress tolerance of *N. flagelliforme*, the role that malonylation plays in drought stress is still unknown. In particular, a better understanding of the impact of Kmal modification on carbon metabolism and photosynthesis is critical to obtain a more comprehensive insight into the drought tolerance mechanisms of *N. flagelliforme*.

In order to investigate the impacts of Kmal on drought stress responses in *N. flagelliforme*, we employed antibody-based affinity enrichment coupled with high-resolution LC-MS/MS analysis. This study represents the first report of the malonylome of a terrestrial cyanobacterium. Our results revealed dynamic changes in malonylation in *N. flagelliforme* under different drought stresses and provided a robust dataset for further exploration of the role that Kmal plays in the stress responses of terrestrial cyanobacteria and other photosynthetic organisms.

## 2. Results

### 2.1. Determination of Kmal in N. flagelliforme

**Figure 1:**
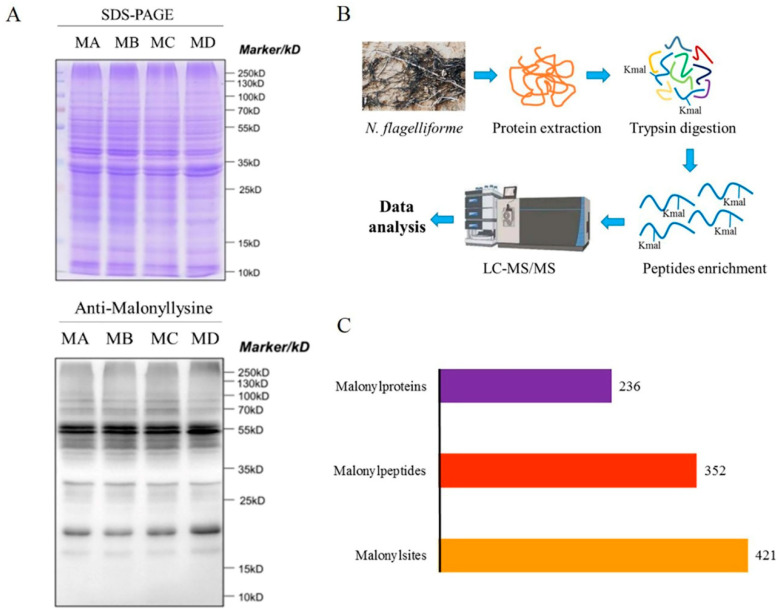
Global identification of Kmal in *N. flagelliforme* under drought stress. (**A**) Identification of Kmal in *N. flagelliforme* during drought stress. SDS-PAGE of proteins isolated from different samples and stained with Coomassie blue. Western blot analysis was performed using a pan anti-malonyllysine antibody. Each channel was loaded with the same amount of protein. The number on the right shows the protein size in kDa. MA, MB, MC, and MD are samples with water loss rates of 0%, 30%, 75%, and 100%, respectively. (**B**) Flowchart for Kmal identification in *N. flagelliforme*. (**C**) Number of malonylated proteins and sites in *N. flagelliforme*.

In order to detect the presence of malonylated protein in *N. flagelliforme*, Western blot analysis was performed using a pan anti-malonyllysine antibody with the proteins of whole cell lysates from different water loss *N. flagelliforme*. A variety of immunoblotting signals with different molecular weights were obtained under different drought stresses (Figure 1A). These results indicated that lysine malonylated proteins were abundant in the colonies and may therefore be involved in the drought stress response. We further analyzed the global malonylome of *N. flagelliforme* under different drought stresses using the LC-MS/MS method (Figure 1B). The mass error distribution was less than 10 ppm (Figure S1). Analysis of the length distribution of the identified peptides indicated that the majority were between 7 and 33 amino acids (Figure S2). A total of 352 Kmal peptides were obtained by mass spectrometry, which contained 421 Kmal sites in 236 unique proteins (Figure 1C). The false discovery rate was found to be below 1% for modified peptides in this study. Details of all identified malonylated peptides are presented in Table S2. Subcellular localization of the identified Kmal proteins showed that most of the malonylated proteins (38%) were found in the cytoplasm and 14% were found in the cytoplasmic membrane. In addition, some malonylated proteins were found in the extracellular space (6%), periplasmic space (2%), and outer membrane (2%) (Figure S3 and Table S3). These findings further indicate that lysine malonylated proteins may be involved in a variety of biological processes.

### 2.2. Analysis of Malonylated Lysine Residues in N. flagelliforme

**Figure 2:**
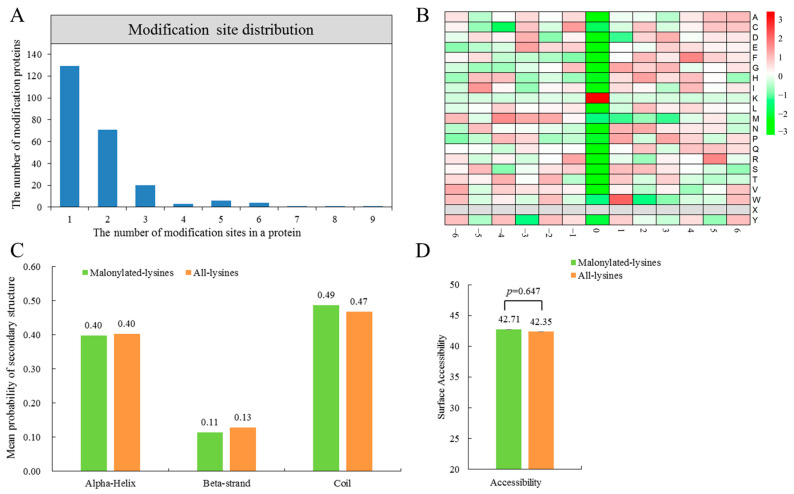
Analysis of Kmal sites. (**A**) The number of Kmal sites for malonylated proteins detected in *N. flagelliforme*. (**B**) Heat map of sequence motifs ranging from −6 to +6 around the lysine malonylation sites. (**C**) Mean probability of malonylated lysine residues and all lysine residues in protein secondary structures (α-helix, β-strand, and coil). (**D**) Surface accessibility of identified malonylation sites.

Malonylation of proteins can occur on one or more lysine residues. It was found that a total of 129 identified malonylated proteins (54.66%) contained a single Kmal site, 71 proteins (30.08%) contained two Kmal sites, and the number of malonylated proteins with three, four, five, and six or more modified sites were 20 (8.47%), 3 (1.27%), 6 (2.54%), and 7 (2.97%), respectively (Figure 2A). Interestingly, alkaline phosphatase was the most malonylated protein and contained nine malonylated sites, which may impact its catalytic activity. The second most highly malonylated protein was the ribulose bisphosphate carboxylase large chain (8 Kmal sites), which is involved in carbon fixation during photosynthesis. In addition, glycerophosphoryl diester phosphodiesterase contained 7 Kmal sites, and is known to be involved in glycerophospholipid metabolism. These results indicated that Kmal impacts proteins involved in a diverse array of biological functions during the drought stress response.

Previous studies demonstrated the modified site preferences for amino acids at specific positions around lysine [10]. Heat map analysis of the amino acid sequences surrounding the malonylated lysine sites did show some biases. For example, a strong bias for phenylalanine (F) at the +2 and +4 position was found (Figure 2B). However, aspartic acid (D), cysteine (C), methionine (M), and tyrosine (Y) were all underrepresented at the +1, −4, +3, and −3 positions, respectively.

The lysine secondary structure localization and relative solvent accessibility of all identified malonylation proteins were analyzed using NetSurfP software (version 1.1). We found that 49% of the Kmal sites were located in unstructured coil regions, 40% in α-helix, and 11% in β-stand regions (Figure 2C). These findings suggest that malonylation is more likely to occur in coil regions rather than α-helix or β-stand regions in proteins. Furthermore, the average relative solvent accessibility of malonylated lysines residues (42.71%) was only slightly higher than that of all lysines (42.35%) (*p* = 0.67); these observation indicated that the malonylation of lysine may not affect the surface properties of the malonylated protein (Figure 2D and Table S4). This result agrees with previous work examining the malonylated lysine residues of maize but differs from other work in *Synechocystis* sp. PCC 6803 [13,25].

### 2.3. Functional Analysis of the Malonylated Proteins in N. flagelliforme during Drought Stress

The differentially expressed peptides were identified with quantitative ratios more than 1.2-fold or less than 0.83-fold, with a *p*-value < 0.05. As shown in Table 1, 11 differentially expressed peptides were up-regulated and 2 were down-regulated in the initial drought (MB vs. MA). In the subsequent drought stages, the differentially accumulated malonylated peptides decreased in both the MC and MD samples. Notably, there were no significantly increased peptides at these time points. These results indicated that malonylated peptides were more strongly down-regulated in response to drought stress as drought severity increased.

To better understand the functions of differentially expressed Kmal proteins in *N. flagelliforme*, GO, domain, and KEGG enrichment analyses were performed (Tables S5–S7). The GO analysis showed that malonylated proteins located in the cell, intracellular, the light-harvesting complex, and the membrane were significantly enriched based on GO cellular component analysis (Figure S4 and Table S5). Consistent with these findings, proteins related to oxidoreductase activity, binding (purine, ribonucleotide, and cofactor), antioxidant activity, and catalytic activity were also highly enriched according to GO molecular function enrichment (Figure S4). Consistently, the proteins involved in photosynthesis, the cellular process, light reaction, the metabolic process, and the oxidation–reduction process were more likely to be malonylated according to GO biological process enrichment in response to drought treatments in *N. flagelliforme* (Figure S4). These findings suggest a potential role of Kmal in photosynthesis and catalytic activity during drought stress in *N. flagelliforme*.

**Figure 3:**
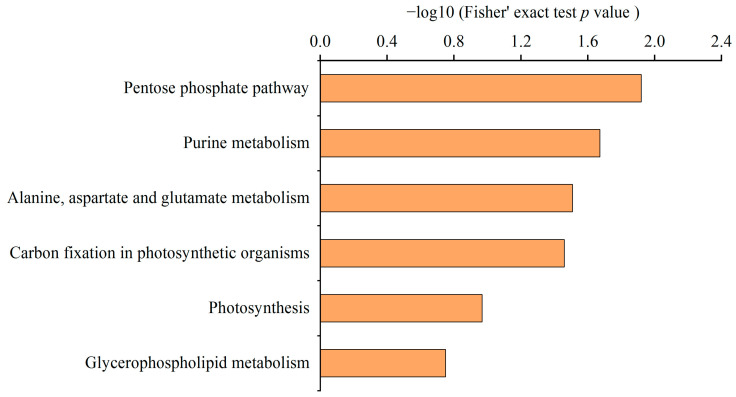
Enrichment analysis of the malonylated proteins based on KEGG pathway in *N. flagelliforme*.

Protein domains were annotated using the InterPro database based on protein sequence alignment, which revealed enrichment in malonylated proteins containing domains associated with the phycobilisome protein, ATP synthase alpha/beta family, AhpC/TSA family, and PRC-barrel domain (Figure S5 and Table S6). Furthermore, KEGG pathway analysis showed that the 23 proteins were enriched in the pentose phosphate pathway, purine metabolism, alanine, aspartate and glutamate metabolism, carbon fixation in photosynthetic organisms, photosynthesis, and glycerophospholipid metabolism (Figure 3 and Table S7). These results suggest that malonylation may regulate an array of proteins associated with the regulation of energy and lipid metabolism.

### 2.4. Protein–Protein Interaction Network

**Figure 4:**
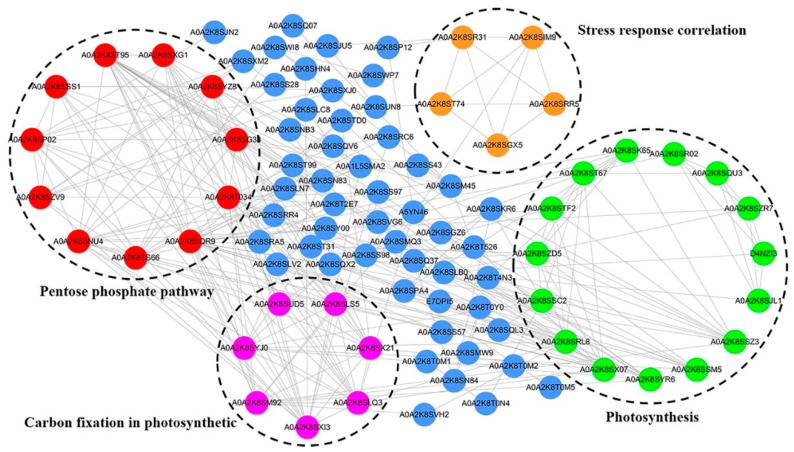
Interaction networks of malonylated proteins in *N. flagelliforme* under drought stress.

In order to better understand the function and regulation of lysine malonylated proteins in *N. flagelliforme*, a protein interaction network was created using Cytoscape (version 3.7.1) based on the STRING database. A total of 109 malonylated proteins were mapped to the protein network, involving 299 identified direct physical interactions. According to the Cytoscape algorithm, four highly interconnected clusters of malonylated proteins were retrieved. The most abundant cluster consisted of 15 photosynthesis-related proteins with 35 edges (Figure 4). Pentose-phosphate-pathway-related Kmal proteins constituted the second major subnetwork, consisting of 11 proteins with 37 edges. In addition, the photosynthetic carbon fixation pathway and stress-response-related proteins were highly interconnected, with seven and five Kmal proteins associated with each, respectively. Overall, these results indicate that malonylation is a key PTM of *N. flagelliforme* that changes in response to drought stress, and is involved in photosynthetic and carbon metabolic pathways.

### 2.5. Conservative Analysis of Lysine Malonylated Proteins in N. flagelliforme

**Figure 5:**
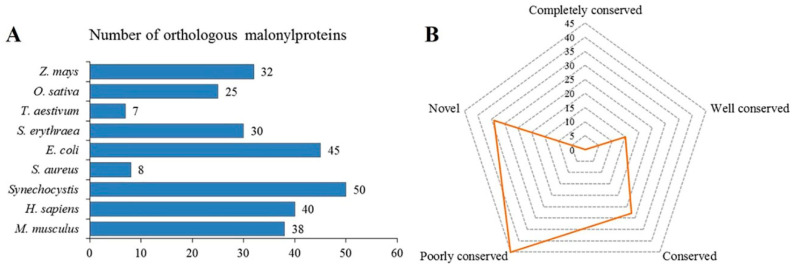
Conservation of lysine malonylated proteins. (**A**) Number of orthologous malonylated proteins in nine reported malonylomes. (**B**) Conservation of malonylated proteins in *Z. mays*, *O. sativa*, *T. aestivum*, *S. erythraea*, *E. coli*, *S. aureus*, *Synechocystis*, *H. sapiens*, and *M. musculus*. The conservation levels were classified as follows: completely conserved, 9 orthologs; well conserved, 6 to 8 orthologs; conserved, 3 to 5 orthologs; poorly conserved, 1 to 2 orthologs; novel, 0 orthologs.

Kmal is also considered to be an evolutionarily conserved PTM in both prokaryotes and eukaryotes [11,15,16]. However, the degree to which specific malonylated protein is conserved in *N. flagelliforme* was previously unknown. To better understand the conservation of this process, we analyzed the orthologs of lysine malonylated proteins in *N. flagelliforme* by employing BLAST to search against nine published organisms with malonylomes: *Staphylococcus aureus*, *Zea mays*, *Triticum aestivum*, *Escherichia coli*, *Saccharopolyspora erythraea*, *Oryza sativa*, *Synechocystis* sp. PCC 6803, *Homo sapiens*, and *Mus musculus*. In total, 275 orthologs of the malonylated proteins in *N. flagelliforme* were obtained in these nine organisms (Figure 5A and Table S8), and 88 differently malonylated proteins have orthologs in *S. aureus* (8 proteins), *Z. mays* (32 proteins), *T. aestivum* (7 proteins), *E. coli* (45 proteins), *S. erythraea* (30 proteins), *O. sativa* (25 proteins), *Synechocystis* (50 proteins), *H. sapiens* (40 proteins), and *M. musculus* (38 proteins), which account for 72.95% (89/122 proteins) of the total differently malonylated proteins in *N. flagelliforme*. We then further classified the conservation of malonyl proteins based on the number of orthologous malonyl proteins in other organisms. This analysis showed that the number of completely conserved proteins (found in all nine species), well conserved proteins (found in six to eight species), conserved proteins (found in three to five species), and poorly conserved proteins (found in one to two species) were 0, 15, 28, and 45 (Figure 5B), respectively. Notably, 34 of the malonylated proteins were identified as novel proteins (zero orthologs) in *N. flagelliforme* in comparison with other organisms. These results indicated that the majority of the malonylated proteins in *N. flagelliforme* were conserved across prokaryotes and eukaryotes, but it also contains unique malonylated proteins with specific functions.

### 2.6. Impact of Lysine Malonylation on Enzymatic Activity

**Figure 6:**
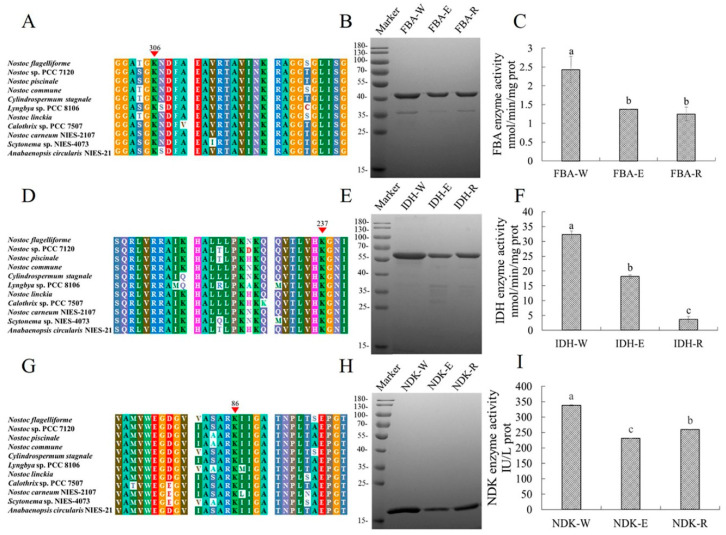
Mutagenesis analysis of malonylated enzymes. (**A**) Multiple sequence alignment of FBA from different cyanobacterial species. (**B**) Coomassie blue staining of purified FBA (FBA-W) and its mutants K306E (FBA-E) and K306R (FBA-R). (**C**) Enzymatic activity of FBA and its mutants K306E and K306R. (**D**) Multiple sequence alignment of IDH from different cyanobacterial species. (**E**) Coomassie blue staining of purified IDH (IDH-W) and its mutants K237E (IDH-E) and K237R (IDH-R). (**F**) Enzymatic activity of IDH and its mutants K237E and K237R. (**G**) Multiple sequence alignment of NDK from different cyanobacterial species. (**H**) Coomassie blue staining of purified NDK (NDK-W) and its mutants K86E (NDK-E) and K86R (NDK-R). (**I**) Enzymatic activity of NDK and its mutants K86E and K86R. The conserved malonylation sites are denoted with red arrows. The different letters above the bars indicate that the means were significantly different (*p* < 0.05).

Since malonylation changes the charge state of lysine from +1 to −1 and adds a malonyl group, it is conceivable that malonylation may change conformation and enzymatic activity to alter cellular metabolic process. Fructose-bisphosphate aldolase (FBA, EC 4.1.2.13) is a pivotal enzyme involved in primary metabolism in plants and cyanobacterium species, which plays important roles in glycolysis and the Calvin cycle, by catalyzing the reversible conversion of fructose-1,6-bisphosphate (FDP) to dihydroxyacetone phosphate and glyceraldehyde-3-phosphate. In this study, one reliable malonylation site (K306) on FBA was identified, and the MS/MS spectrum of the modified peptide revealed the exact site of malonylation (Figure S6). According to multi-sequence alignment analysis, the K306 modification site of FBA was highly conserved in cyanobacteria (Figure 6A), suggesting that this lysine residue might play an important role in FBA functions. To further evaluate the potential regulatory effect of malonylation at this site on FBA, we replaced lysine residue (K306) with arginine or glutamic acid to obtain the mutants FBA-E and FBA-R, respectively. DNA sequencing and mass spectrometry were performed to confirm that all mutants were correctly constructed (Figure S7). Next, we purified and measured the enzymatic activity of wild-type FBA and its mutants and found that both FBA-E and FBA-R had decreased activity compared to the wild type, with no significant difference seen between the two mutants (*p* < 0.05) (Figure 6B,C). These findings suggest that malonylation may alter the enzymatic activity of FBA.

We also found that isocitrate dehydrogenase (IDH, EC 1.1.1.42) is malonylated. IDH catalyzes the oxidative decarboxylation of isocitrate to 2-oxoglutarate with the production of the reduced coenzyme NADPH, which is a key rate-limiting enzyme in the TCA cycle. We identified the malonylated K237 position in this protein and found that it was highly conserved in different cyanobacteria species (Figure 6D). To assess the impact of this malonylation site, we generated IDH mutants and verified them via sequencing and MS (Figure S8). When this site was converted to E, IDH-E activity decreased significantly compared with WT, and when this site was mutated to R, IDH-R activity decreased even more significantly (Figure 6E,F). The results revealed the importance of the malonylated K237 site for IDH activity.

In addition, nucleoside diphosphate kinase (NDK, EC 2.7.4.6) can reversibly transfer phosphates from ATP to cognate nucleoside diphosphates to sustain the balance between ATP and other nucleoside triphosphates, and mediates redox balance by regulating upstream intracellular nucleotide pools [26]. A highly conserved malonyl site K86 in NDK was observed in this study, and we generated mutants that converted this modified lysine residue to glutamic acid and arginine (Figure 6G and Figure S9). In contrast to WT, the mutation of Lys86 to E and R reduced its enzymatic activity (*p* < 0.05) (Figure 6H,I), suggesting that Kmal was a potential regulated way to alter the enzymatic activity of NDK.

### 2.7. Physiological Response to Drought Stress

**Figure 7:**
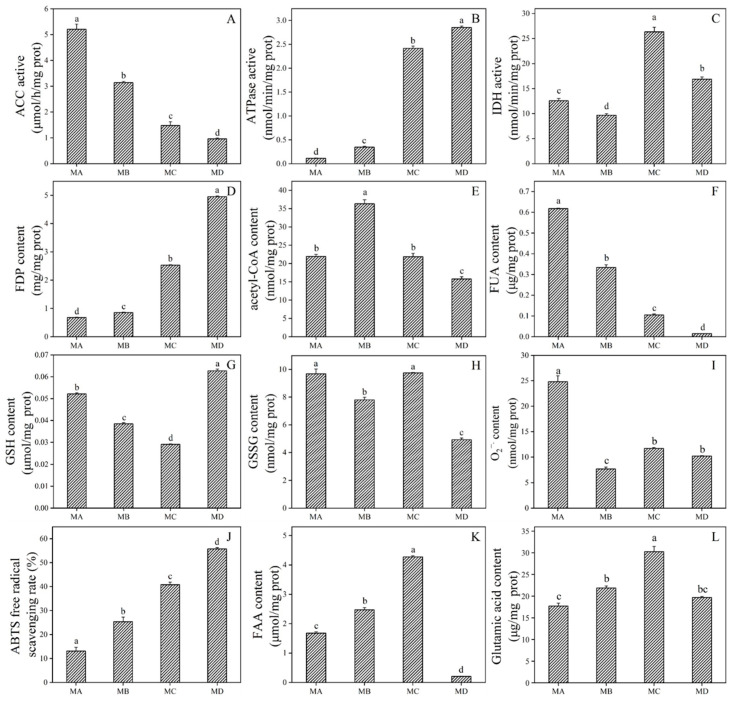
Influence of drought stress on metabolite contents and key enzyme activities of *N. flagelliforme*. (**A**) acetyl-CoA carboxylase (ACC), (**B**) ATP synthase (ATPase), and (**C**) isocitrate dehydrogenase (IDH) activity; (**D**) fructose-1,6-diphosphate (FDP), (**E**) acetyl-CoA, (**F**) fumaric acid (FUA), (**G**) glutathione (GSH), (**H**) glutathione disulfide (GSSG), and (**I**) superoxide anion (O_2_^−•^) content; (**J**) free radical scavenging rate; (**K**) free fatty acids (FFA), and (**L**) glutamic acid content. The values are shown as mean ± standard error of three replicates and different letters represent significantly different values (*p* < 0.05).

ACC is known to catalyze malonyl-CoA production, and we found that its activity decreased during drought stress (Figure 7A). Next, we determined the enzymatic activity and metabolites in the metabolic process of malonylated proteins in drought-stressed *N. flagelliforme*. Three subunits of ATPase were malonylated, and its activity was found to increase during drought stress. The activity of IDH, a key enzyme in the TCA cycle, increased during severe drought stress (water loss 75% and 100%) (Figure 7C). In addition, we observed a significant increase in FDP content in glycolysis, with opposite changes in acetyl-CoA and fumaric acid content in the TCA cycle under drought treatment (Figure 7D–F). We measured the contents of two metabolites involved in ROS clearance related to malonylated GR and GST: GSH and GSSG (Figure 7G,H). The results showed that GSH content first decreased and then increased, while GSSG content decreased significantly with drought. This in turn led to a decreased O_2_^−•^ content and an increased free radical scavenging rate, likely due to the reaction catalyzed by the malonyl regulatory enzymes (Figure 7I,J). As mentioned above, KEGG enrichment analysis indicated that malonylated protein was involved in glycerophospholipid metabolism, and lipolysis produces FFA. It was also found that the content of FFA increased significantly under drought stress (Figure 7K). Additionally, some enzymes related to amino acid synthesis were malonylated. Our measurement of amino acid content indicated that during drought stress, the levels of glutamic acid, serine, glycine, and asparagine increased, the content of glutamine decreased, and the content of tryptophan did not change (Figure 7L and Figure S10).

## 3. Discussion

### 3.1. Photosynthesis

**Figure 8:**
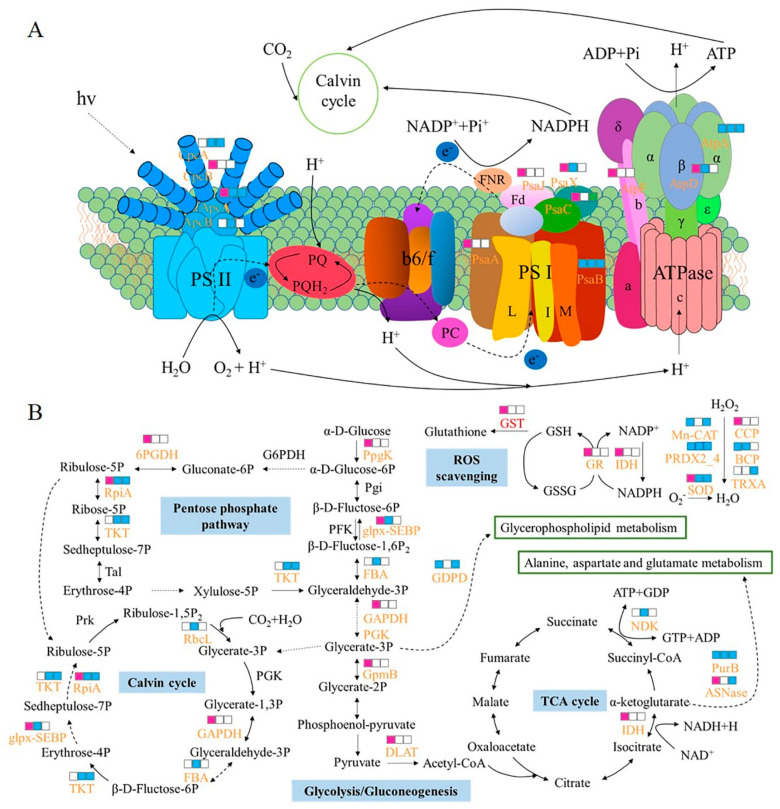
Working scheme of Kmal events involved in carbon metabolism, photosynthesis, and ROS scavenging in *N. flagelliforme* during drought stress. (**A**) Illustrations of malonylated proteins involved in the photosynthesis process. (**B**) Overview of the impact of the Kmal process on carbon metabolism and antioxidant pathways of *N. flagelliforme*. The identified malonylated proteins are marked in orange. The small squares arranged from left to right indicate the changes in the level of malonylation modification in MB, MC, and MD groups compared with the control group. White indicates no significant difference, pink indicates up-regulation, and blue indicates down-regulation. Solid arrows represent a direct process and dashed arrows represent indirect processes.

Photosynthesis is the energy source for all physiological activities of photosynthetic organisms, and Kmal has been found to play a critical role in this process in both higher plants and cyanobacteria [12,14]. The malonylation levels of phycocyanin subunits α and β (CpcA and CpcB) and allophycocyanin subunits α and β (ApcA and ApcB) in the light-harvesting system were found to decrease during drought, which likely hinders the transmission of light energy to reduce photosynthesis. Similar findings have been observed in alfalfa pods during drought stress [27]. This is also in keeping with our previous finding that photosynthesis is reduced in *N. flagelliforme* under drought stress [18]. PsaA and PsaB form central heterodimers as the core of PSI [28]. We found that the malonylation level of PsaA was up-regulated in the early stage of drought stress, while that of PsaB was significantly reduced (Figure 8A). Moreover, the malonylation levels of PsaC, PsaJ, and PsaX (1.59-fold) all increased at 30% water loss, but then decreased with further drought stress (Figure 8A). Similar to the Kmal seen in *Synechocystis* sp. 6803, the malonylation of these PSI subunits may be involved in photosynthetic complex assembly or disassembly, as well as energy transfer processes [12]. We also found that the malonylation levels of AtpA, AtpD, and AtpF were decreased during drought, which likely affected the activity of ATPase. Further measurement showed that ATPase activity significantly increased during drought stress (Figure 7B). Our previous results showed that ATP content increased when water loss was 100% in *N. flagelliforme* [24], which may be due to the increase in ATP synthase activity to generate energy for other physiological activities to adapt to drought stress. These findings indicate that malonylation might block the reception and transmission of light energy to reduce photosynthesis, and accumulate ATP by increasing enzymatic activity to adapt to drought stress.

The Calvin cycle is the primary pathway of photosynthetic carbon fixation, and drought stress affects the expression and activity of metabolic enzymes in this pathway. Ribulose-1,5-bisphosphate carboxylase/oxygenase (RuBisCO) plays an important role in CO_2_ assimilation during carbon fixation, and many studies have shown that the activity of RuBisCO decreases during drought [29], the same as in *N. flagelliforme* [24]. In this study, eight Kmal sites were identified in the large chain of RuBisCO (RbcL), and the malonylation of RbcL was reduced in response to drought stress (Table S2 and Figure 8B). The change likely acts to inhibit carbon fixation during drought. Interestingly, D-fructose 1,6-bisphosphatase class 2/sedoheptulose 1,7-bisphosphatase (glpx-SEBP), a unique enzyme in cyanobacterial cells that has both fructose-1,6-bisphosphatase and sedoheptulose-1,7-bisphosphatase (SBPase) activities, were malonylated. It has been reported that overexpression of the cyanobacterial *FBP/SBPase* gene in the chloroplasts of transgenic tobacco and lettuce enhanced the CO_2_ assimilation rate and increased plant biomass [30,31]. In this study, malonylation levels of glpx-SEBP were up-regulated at 30% water loss and then decreased with more severe drought (Figure 8B), which may hinder the regeneration of CO_2_ and lead to a reduction in photosynthesis.

### 3.2. Central Carbon Metabolism

Studies have shown that the feedback exerted by malonylation on glycolysis flux could redirect glucose away from oxidation in glycolysis towards glycogen synthesis or the pentose phosphate pathway [32]. We found that the content of fructose-1,6-diphosphate (FDP) significantly increased under drought stress (Figure 7D), which is consistent with the previously reported results of glucose and glycogen accumulation in *N. flagelliforme* under drought stress [24]. In addition, fructose-1,6-diphosphatase (FBP) is the rate-limiting enzyme of gluconeogenesis, and it was found that FBP activity increased significantly with drought stress in our previous study [24], suggesting that the gluconeogenesis pathway was active. It is worth noting that the pyruvate dehydrogenase complex (PDHc), which converts pyruvic acid into acetyl-CoA, controls the entry of carbon into the TCA cycle [33]. Studies have shown that the activity of PDHc is positively correlated with the production of acetyl-CoA, and increased activity of the PDHc enzyme complex redirects pyruvate metabolism to the TCA cycle [34]. In this study, the change in the malonylation level of DLAT was consistent with the content of acetyl-CoA, which increased at 30% water loss, and then decreased significantly (Figure 7E). This is very likely due to the effects of Kmal on DLAT, which is a subunit of PDHc, thereby decreasing PDHc activity and reducing the carbon source flow to the TCA cycle during severe drought (75% and 100% water loss). It has been observed in *G. uralensis* that the reduction in PDHc activity inhibits the TCA cycle [35]. We further observed that fumaric acid, an intermediate metabolite involved in the TCA cycle, significantly decreased with drought (Figure 7F), which provides further evidence of TCA inhibition under drought stress. Together, our results showed that Kmal weakens the flux of glycolysis to the TCA cycle by controlling the activity of metabolic enzymes, activates the gluconeogenesis pathway, and causes the accumulation of osmotic-adjusting substances such as glucose and sucrose in response to drought stress.

Furthermore, glucose-6-phosphate is converted into ribulose-5-phosphate by 6-phosphogluconate dehydrogenase (6PGDH), which is accompanied by the production of NADPH. We found that its malonylation level was up-regulated, and correspondingly, the enzyme activity of 6PGDH was also significantly increased in *N. flagelliforme* [24], which contributed to the accumulation of reducing power to adapt to severe drought. This result further supports the idea that malonylation is involved in the response of energy metabolism to drought stress by regulating the activity of enzymes in carbon metabolic pathways.

### 3.3. Antioxidant Defense

The antioxidant defense system plays a key role in the responses to drought stress of plants, limiting the damage to the repairable level and maintaining physiological integrity during the drying duration [36]. Notably, we observed that malonylated proteins were significantly enriched when related to the ROS scavenging system (Figure 8B). We found that the malonylation level of SOD increased at the initial stage of drought stress (1.65-fold) but decreased as drought became more severe (75% and 100% water loss), which was in line with the decrease in SOD activity during drought in *N. flagelliforme* [24]. It may be that malonylated SOD acts on a certain degree of drought stress and keeps low activity when damaged by excessive ROS during drying. These findings indicate that the malonylated antioxidant enzymes played a major role in the removal of ROS at the early stage of drought stress.

Glutathione (GSH) can be oxidized to GSSG by H_2_O_2_, and the resulting GSSG is reduced in turn to GSH by GR, which is accompanied by significant changes in redox potential [37]. In this study, the content of GSH increased significantly during severe drought, while the content of GSSG decreased significantly (Figure 7G,H). Hence, the accumulation of GSH in *N. flagelliforme* under drought stress may mediate the scavenging of ROS. These findings indicate that the antioxidant enzymes of *N. flagelliforme* play a metabolic oxidative detoxification role in the early stage of drought stress, while the antioxidant substances play a role in the late stage of drought stress.

### 3.4. Lipid Metabolism and Amino Acid Biosynthesis

As the donor of the malonyl group, malonyl-CoA can be synthesized by acetyl-CoA carboxylase (ACC) and consumed by malonyl-CoA decarboxylase (MCD) [9,38]. We found that ACC enzymatic activity decreased significantly during drought (Figure 7A), which may be the main reason for the decrease in the malonylation level of *N. flagelliforme*. In addition, it has been shown that mouse *ACC2* knockout mutants have increased fatty acid oxidation [9]. This suggests that low levels of ACC promote the oxidative decomposition of fatty acids, and consistent with this result, we observed the content of FFA increased significantly in *N. flagelliforme* during drought (Figure 7K). Notably, we found that glycerophosphoryl diester phosphodiesterase, a protein involved in glycerophospholipid metabolism, was significantly malonylated at seven sites (Table S2). Among them, the malonylated K332 site is in close proximity to the metal binding active site E330 when compared against the NCBI database, which indicates that the malonylation of this residue may be important in regulating protein function. Together, these results further demonstrate that malonylation is closely related to lipid metabolism and participates in the response of *N. flagelliforme* to drought stress by regulating lipid degradation.

Notably, lysine malonylated proteins have been found to be significantly enriched in functions associated with amino acid degradation and synthesis in eukaryotic and prokaryotic cells [10,14,15], indicating that malonylation may be involved in regulating protein metabolism. In this work, KEGG analysis indicated that malonylated proteins were significantly enriched for alanine, aspartate, and glutamate metabolism (Figure 3). Enzymes involved in the biosynthesis of amino acids have also been found to be malonylated, all of which contain more than one malonylation site (Table S2). We further assessed the content of amino acids in *N. flagelliforme* under drought stress and found that the contents of glutamic acid, serine, glycine, and asparagine increased during drought stress (Figure 7L and Figure S10). Previous work has shown that the accumulation of free amino acids under drought stress enhances plant tolerance through osmotic adjustment [39,40]. These results suggest that Kmal may affect the activities of metabolic enzymes in the biosynthetic pathway of amino acids, and then regulate the changes in amino acids’ content in *N. flagelliforme* to enhance drought tolerance.

**Figure 9:**
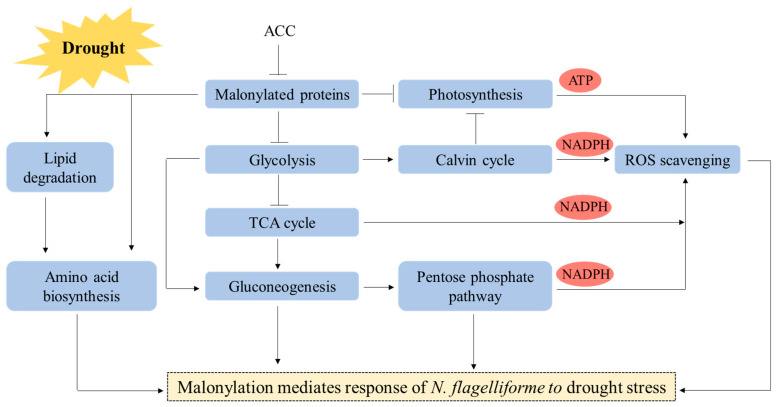
Probable regulation mechanism of Kmal modification in *N. flagelliforme* under drought stress.

In conclusion, a total of 236 malonylated proteins containing 421 unique modification sites were identified and found to be localized to different cell compartments. This work represents the first analysis of the malonylome of terrestrial cyanobacteria. We found that Kmal changes during drought stress weakened the flux of glycolysis to the TCA cycle and activated the gluconeogenesis pathway. In addition, malonylation hindered the reception and transmission of light energy and inhibited carbon fixation, resulting in a decrease in photosynthesis. Malonylated antioxidant enzymes played a role in the early stage of drought stress, while redox homeostasis was maintained mainly by adjusting antioxidant levels during severe drought stress. We also found that malonylation was involved in lipid degradation and amino acid biosynthesis in response to drought stress. Taken together, our findings provide a useful resource for further studies of the biological role of Kmal in abiotic stresses, especially drought. Figure 9 suggests the possible mechanism of drought stress of terrestrial cyanobacteria related to Kmal modification.

## 4. Materials and Methods

### 4.1. Materials and Stress Treatments

*Nostoc flagelliforme* (Berkeley & Curtis) Bornet & Flahault samples were collected from the Helan mountain east region, Ningxia, China. The culture conditions and water loss experiments were carried out as previously described [18]. The samples MA, MB, MC, and MD were obtained at water loss 0%, 30%, 75%, and 100%, respectively. After harvesting, the samples were stored at −80 °C until further analyses.

### 4.2. Protein Extraction

The total protein extraction was performed according to the improved trichloroacetic acid (TCA) method described in our previous report [23]. The protein concentration was measured using the standard Bradford method [41], and protein abundance was observed by Coomassie brilliant blue staining and decolorization.

### 4.3. SDS-PAGE and Western Blot Analysis

The extracted proteins from each group of samples were separated by SDS-PAGE electrophoresis, and then transferred to a polyvinylidene difluoride membrane. The membrane was first incubated with pan anti-malonyllysine antibody (PTM-902, PTM Biolabs, Chicago, IL, USA) at 1:1000 (*v*/*v*) dilution. Goat anti-Mouse IgG peroxidase conjugated secondary antibody (31430, Thermo pierce, Rockford, IL, USA) was used at a 1:5000 (*v*/*v*) dilution. The proteins were then detected with an enhanced chemiluminescence immunoblotting detection kit (Advansta, San Jose, CA, USA).

### 4.4. Trypsin Digestion and Affinity Enrichment

Protein trypsin digestion and enrichment was carried out as described by Wang et al. [23]. The dissolved peptides were incubated with anti-Kmal antibody beads (PTMScan malonyl-lysine Motif Kit, Cell Signaling Technology, Danvers, MA, USA) to enrich the malonylated peptides. The supernatant was then collected and desalted with C18 STAGE Tips following manufacturer’s instructions [24].

### 4.5. LC-MS/MS Analysis

The enriched malonylated peptides were separated by an Easy nLC system (Thermo Fisher Scientific, Waltham, MA, USA). In brief, peptides were dissolved in 0.1% formic acid, and then loaded onto sample column (Thermo Scientific Acclaim PepMap100, 100 μm × 2 cm, nanoViper C18) with an automatic sampler. The samples were separated by an analytical column (Thermo Scientific EASY column, 10 cm, 75 μm ID, 3 μm, C18-A2) with a flow rate of 300 nL min^−1^. The peptide separation gradient parameters were set according to previously described methods [14]. The separated peptides were then analyzed by a Q Exactive mass spectrometer (Thermo Fisher Scientific, Waltham, MA, USA). For MS scans, the scanning range of the precursor ion was 300–1800 *m*/*z*. The resolution of the MS1 spectrum was 70,000 at 200 *m*/*z*, the target of AGC (automatic gain control) was 1 × 10^6^, the Maximum IT was 50 ms, and the dynamic exclusion was 60.0 s. The mass charge ratios of peptides and polypeptide fragments were determined as described by Li [42].

### 4.6. Data Analysis

MaxQuant software was used to process MS/MS data, including peak list generation of raw MS data and recalibration of precursor mass for protein identification and quantification [38]. Trypsin was specified as the cleavage enzyme, and a maximum of three missed cleavage sites were permitted. The *N. flagelliforme* protein database (uniprot_Nostoc_flagelliforme_10681_20201211.fasta) concatenated with a reverse decoy database was utilized for database searches. The threshold of false detection rate of proteins, peptides, and modified sites was fixed at 0.01. For all other parameters, MaxQuant was set to default values [14].

### 4.7. Bioinformatics Analysis

PSORTb 3.0 software was used to predict the subcellular localization of all malonylated proteins identified [12], while NetSurfP (version 1.1, DTU Health Tech, Copenhagen, Denmark) was used for the prediction of protein secondary structure [12,43]. The obtained malonylated proteins were annotated using Gene Ontology (GO) terms derived from the UniProt-GOA database, as described previously [44]. The Kyoto Encyclopedia of Genes and Genomes (KEGG) database was used to annotate protein pathways [45]. The functional domains of identified proteins were annotated by using the InterPro domain database [46]. The protein–protein interactions of the identified malonylated proteins were predicted by searching against the STRING database (version 11.5, https://cn.string-db.org/) and then visualized using Cytoscape (version 3.7.1, National Resource for Network Biology, La Jolla, CA, USA) [15]. The conservation of the identified proteins was performed by BLASTP [11].

### 4.8. Cloning, Mutagenesis, and Purification of FBA, IDH, and NDK Proteins

The wild-type fructose-bisphosphate aldolase, class I (*FBA*), NADP-isocitrate dehydrogenase (*IDH*), and nucleoside diphosphate kinase (*NDK*) genes were amplified by using the primers FBA-F, FBA-R, IDH-F, IDH-R, NDK-F, and NDK-R, with the genomic DNA of *N. flagelliforme* as the template (Table S1). The mutant sequences of FBA-E, FBA-R, IDH-E, IDH-R, NDK-E, and NDK-R were synthesized by Tsingke Biotechnology Co., Ltd., Nanjing, China. The polymerase chain reaction product was inserted into a pMD18-T plasmid (Takara Bio, Shiga, Hikone, Japan) and confirmed by sequencing. The plasmids containing wild-type or mutated genes were double digested with BamH I and Xho I. Then, the digested fragments were inserted into pET28a expression vectors to generate recombinant expression vectors. The constructed plasmids were transformed into *E. coli* BL21 (DE3) for protein expression.

The recombinant strains were cultured in Luria–Bertani medium at 37 °C with 50 µg mL^−1^ kanamycin to an OD_600_ of 0.6–0.8. Protein expression was then induced by treatment with 1 mM isopropyl-b-D-thiogalactoside at 37 °C. The collected cells were then washed with PBS. To obtain soluble NDK protein, the pellets were resuspended in binding buffer (20 mM Phosphate Buffer (including Na_2_HPO_4_ and NaH_2_PO_4_), 500 mM NaCl, 10 mM Imidazole, pH 7.4), and disrupted by ultrasonication. The supernatant was purified with affinity Ni^2+^ protein purification beads containing a His-tag (Beaver, Suzhou, China). To obtain the insoluble proteins FBA and IDH, samples were sonicated in PBS buffer. Inclusion body proteins were solubilized in denaturing solution (8 M urea, 100 mM NaCl, 50 mM Tris, 1 mM DTT, and 1 mM EDTA, pH 8.0) at 4 °C. The supernatant was loaded onto affinity Ni^2+^ protein purification beads. The elution was transferred to a dialysis bag and sealed. The renaturation solution (100 mM NaCl, 50 mM Tris, 1 mM DTT, and 1 mM EDTA, pH 8.0) was then added at a ratio of 1:10, with urea concentration gradients of 4 M, 2 M, 1 M, 0.5 M, 0.25 M, and 0 M. Additionally, 0.5 M arginine, 2 mM GSH, 0.2 mM GSSG, and 10% glycerol were added into the renaturation solution to help the denatured protein fold correctly during renaturation. All purified proteins and renatured proteins were examined by SDS-PAGE.

### 4.9. Enzymatic Activities and Metabolite Assays

The activities of FBA, IDH, ATP synthase (ATPase), and acetyl-CoA carboxylase (ACC) were measured according to the instructions for the kit from Suzhou Michy Biotechnology Co., Ltd. (Suzhou, China). Briefly, about 0.1 g of the sample was weighed, and 1 mL of extract solution was added for ice bath homogenization. The solution was centrifuged at 4 °C, 8000× *g* for 10 min, then the supernatant was taken and placed on ice for testing. The activities of NDK were determined using an assay kit (Jingmei Biological Technology Co., Ltd., Jiangsu, China) according to the manufacturer’s instructions. In brief, the purified enzyme was added into the test wells of the microtitration plate coated with the antibody and then combined with horseradish peroxidase (HRP)-labeled detection antibody. Tetramethylbenzidine substrate solution was added, and the reaction was terminated with the sulfuric acid solution. The absorbance at 450 nm was measured by spectrophotometry. The contents of glutathione (GSH), glutathione disulfide (GSSG), acetyl-CoA, fructose-1,6-diphosphate (FDP), free fatty acids (FFA), and superoxide anion (O_2_^−•^) as well as antioxidant capacity were determined by using a 2,2′-azinobis-3-ethylbenzotiazoline-6-sulfonic acid (ABTS) radical scavenging capacity assay and ferric reducing antioxidant potential (FRAP) assay produced by Suzhou Michy Biotechnology Co., Ltd. In addition, the contents of glutamic acid, serine, glutamine, asparagine, glycine, tryptophan, and fumaric acid (FUA) were measured using high-performance liquid chromatograph.

### 4.10. Statistical Analysis

All measurements were performed with three independent biological replicates. The results are shown as means ± standard error (SE). The differential comparisons among groups were tested by one-way analysis of variance with SPSS 17.0 (IBM, New York, NY, USA). The differences were considered statistically significant at *p* < 0.05.

## Figures and Tables

**Table 1:** Quantitative statistical result of malonylated peptides in *N. flagelliforme*.

Comparisons	Significant Changes in Abundance		Consistent Presence/Absence Expression Profile	
	Increased	Decreased	Increased	Decreased
MB vs. MA	11	2	118	26
MC vs. MA	0	18	1	37
MD vs. MA	0	10	7	33

## Data Availability

All data are available in the manuscript or the supplementary materials.

## Supplementary Materials

The supporting information can be downloaded at: https://www.mdpi.com/article/10.3390/ijms24098446/s1.
