# Supplementary material for: The Enzyme Lysine Malonylation of Calvin Cycle and Gluconeogenesis Regulated Glycometabolism in Nostoc flagelliforme to Adapt to Drought Stress
Source: Int J Mol Sci. 2023 May 8;24(9):8446. doi: 10.3390/ijms24098446 (PMC10179182; doi:10.3390/ijms24098446)
Supplement: Supplementary file 1 [file ijms-24-08446-s001.zip › ijms-2338604-Supplementary Figure.pdf]

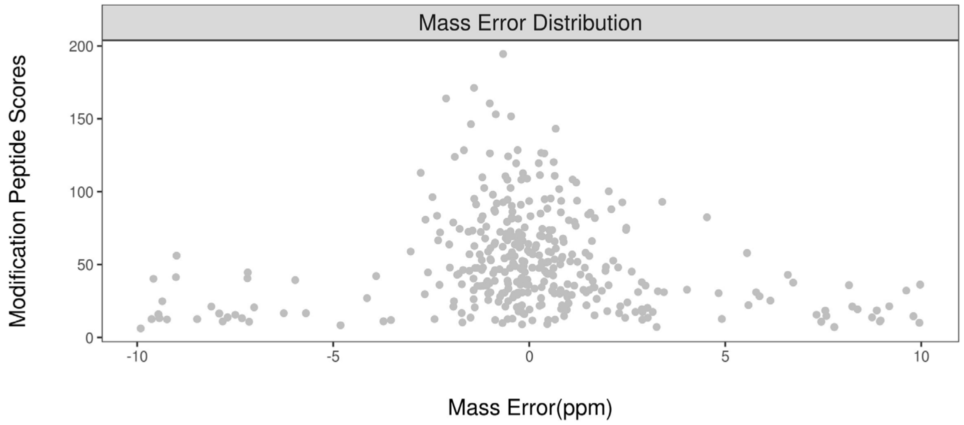

Figure S1. Distribution of peptide mass error based on the malonylation modification of *N. flagelliforme*.

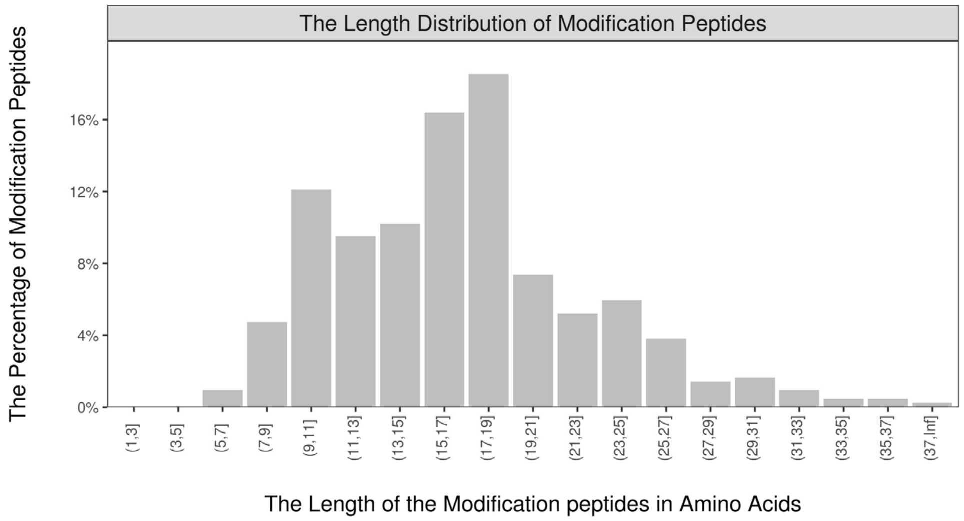

Figure S2. The length distribution of malonylated peptides of *N. flagelliforme*.

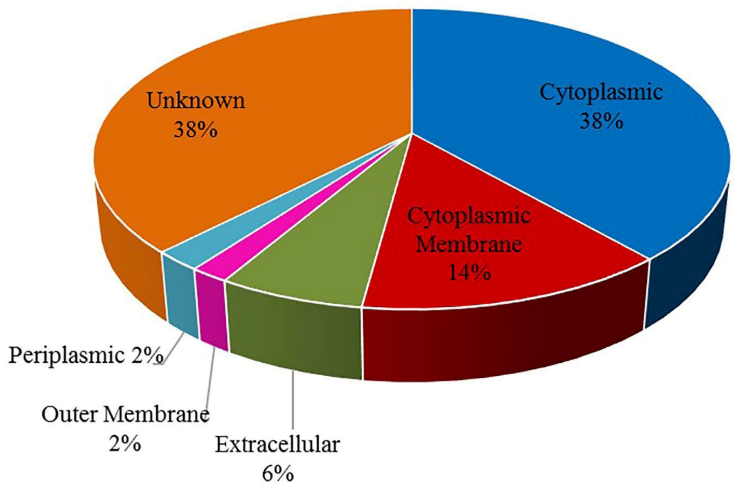

Figure S3. Analysis the subcellular localizations of the modified proteins in *N. flagelliforme*.

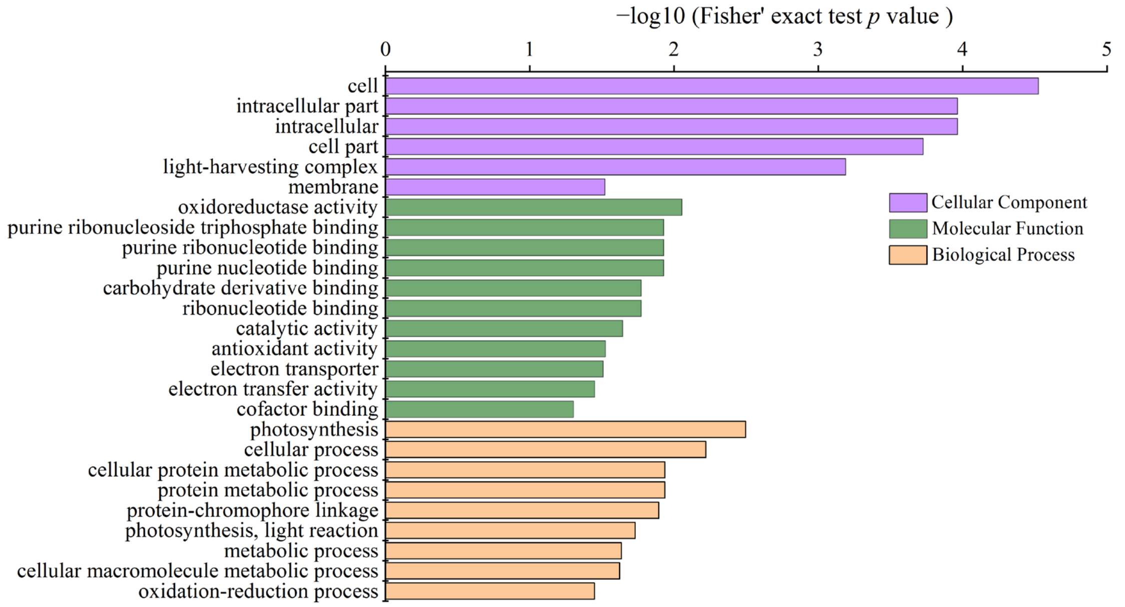

Figure S4. Functional enrichment based on GO molecular function, cellular components and biological process.

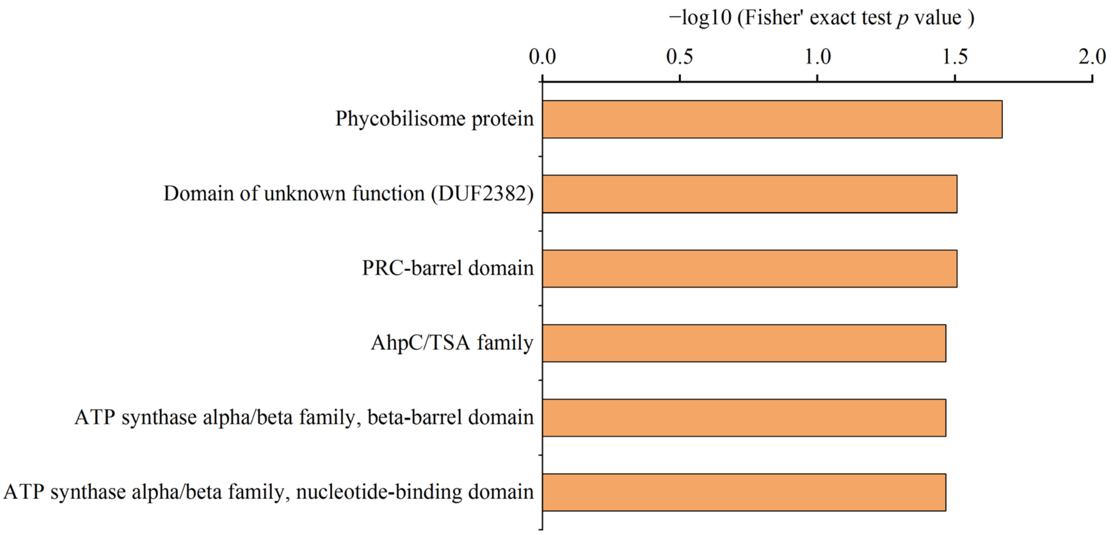

Figure S5. Domain functional enrichment of the identified proteins.

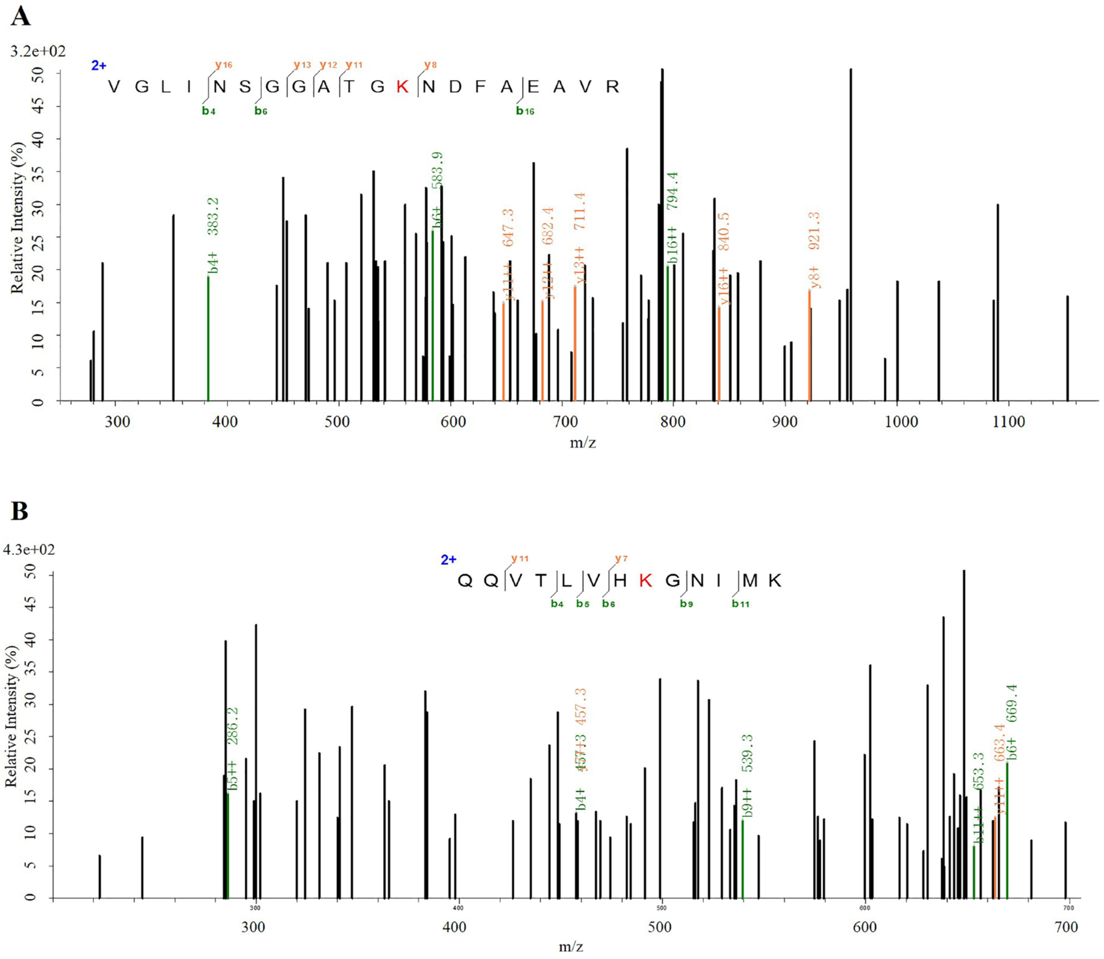

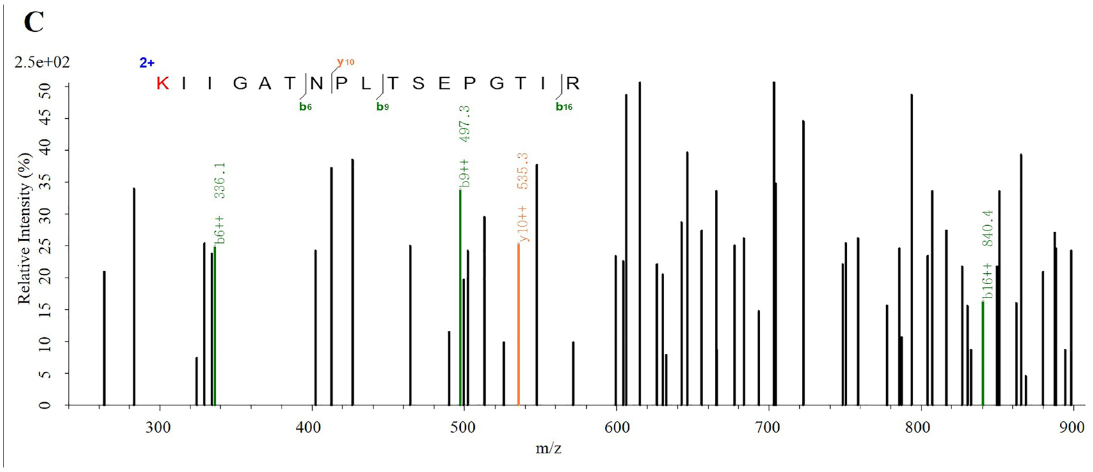

Figure S6. Profiling of identified malonylation sites on FBA, IDH and NDK in *N. flagelliforme* under drought stress. (A) The representation of the corresponding MS/MS spectra assigned to the malonylated peptides from FBA. Malonylpeptide VGLINSGGATGK(mal)NDFAEAVR of FBA (A0A2K8SZV9). (B) The representation of the corresponding MS/MS spectra assigned to the malonylated peptides from IDH. Malonylpeptide QQVTLVHK(mal)GNIMK of IDH (A0A2K8SM45). (C) The representation of the corresponding MS/MS spectra assigned to the malonylated peptides from NDK. Malonylpeptide K(mal)IIGATNPLTSEPGTIR of NDK (A0A2K8T2E7). In the sequence of peptide, the site of modification is indicated as (mal) in red color, green color and yellow color represent the b ions and y ions, respectively.

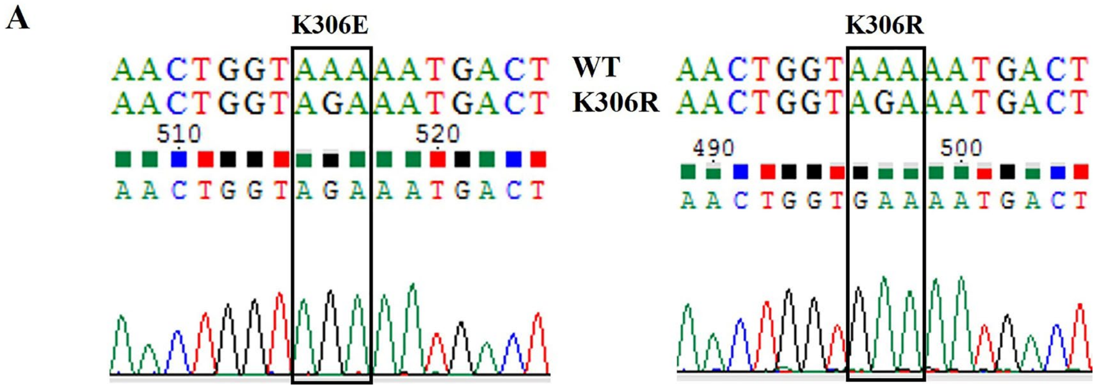

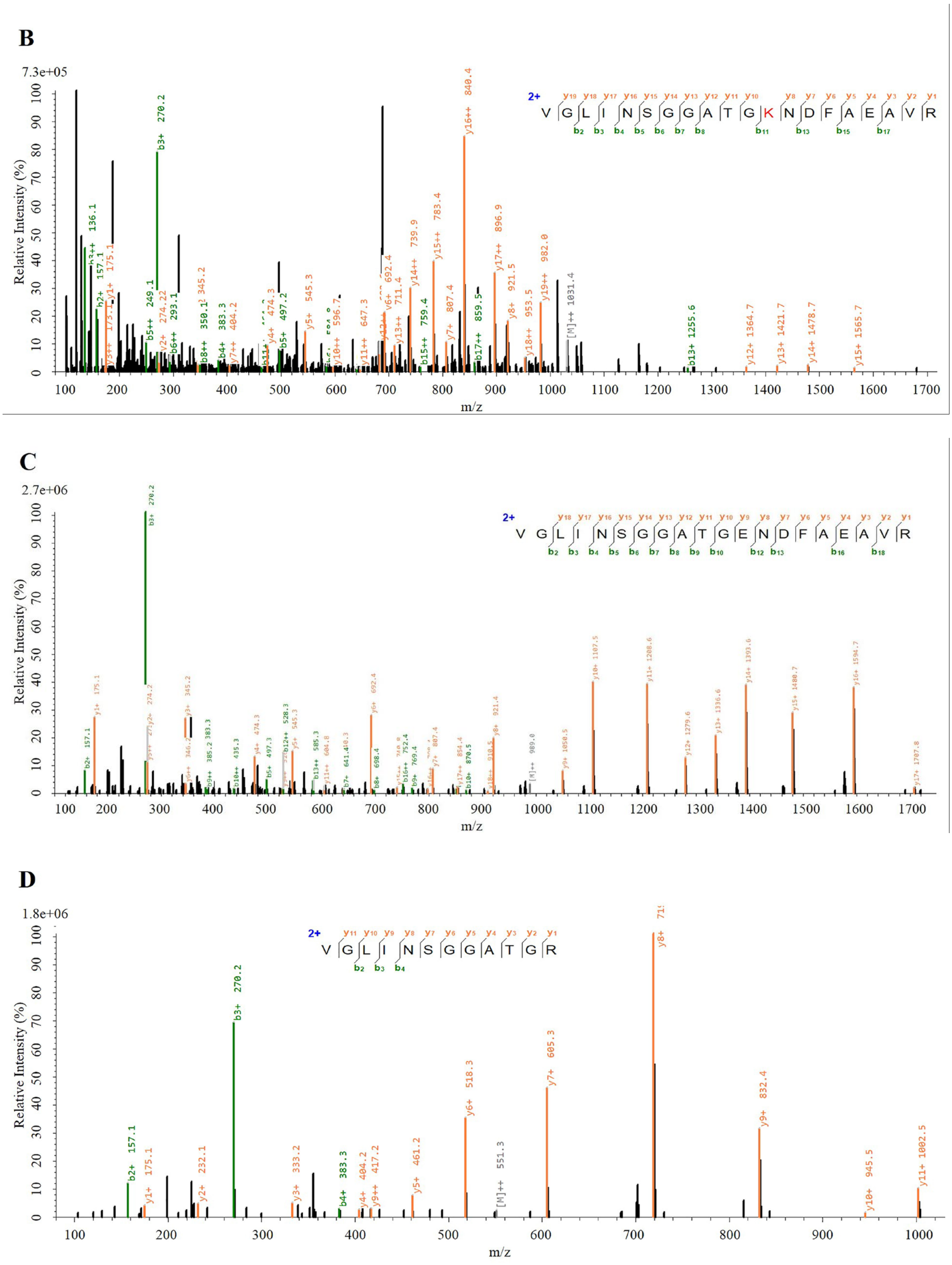

Figure S7. Mutations of FBA were verified by DNA sequencing and MS searches. (A) DNA sequencing verification site mutation. (B) K306 malonylation site in wild-type confirmed by MS analysis. (C) The K306E mutation site in the FBA-E mutant was confirmed by MS analysis. (D) The K306R mutation site in the FBA-R mutant was confirmed by MS analysis.

A

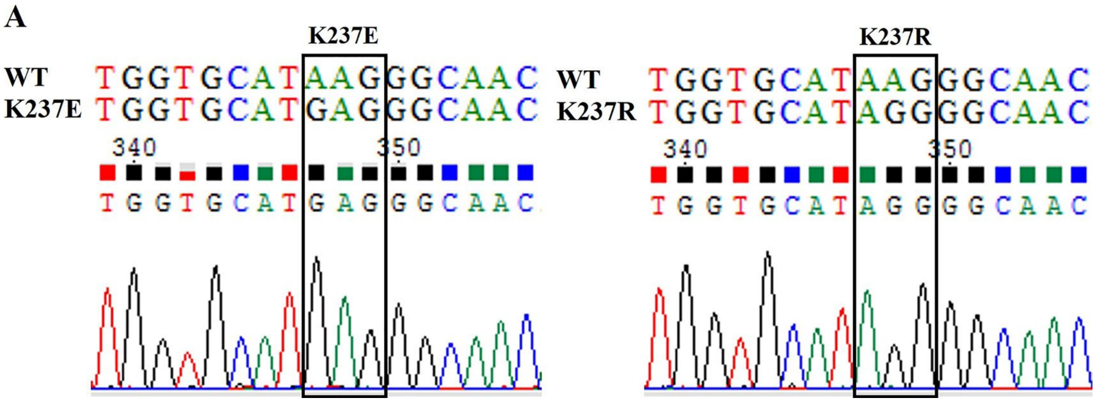

K237E
K237R
WT TGGTGCATAAGGGCAAC
K237E TGGTGCATGAGGGCAAC
WT TGGTGCATAAGGGCAAC
K237R TGGTGCATAGGGGCAAC
340
350
TGGTGCATGAGGGCAAC
TGGTGCATAGGGGCAAC

B

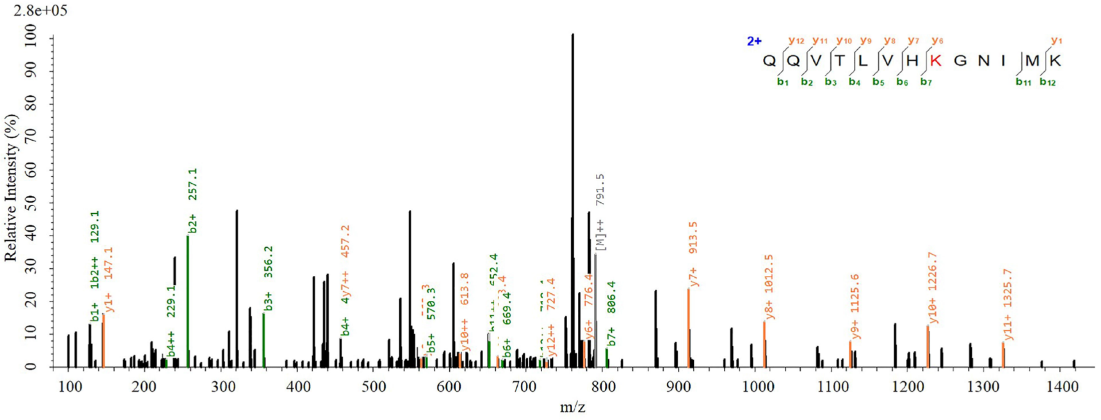

2.8e+05
Relative Intensity (%)
m/z
2+
Q Q V T L V H K G N I M K

C

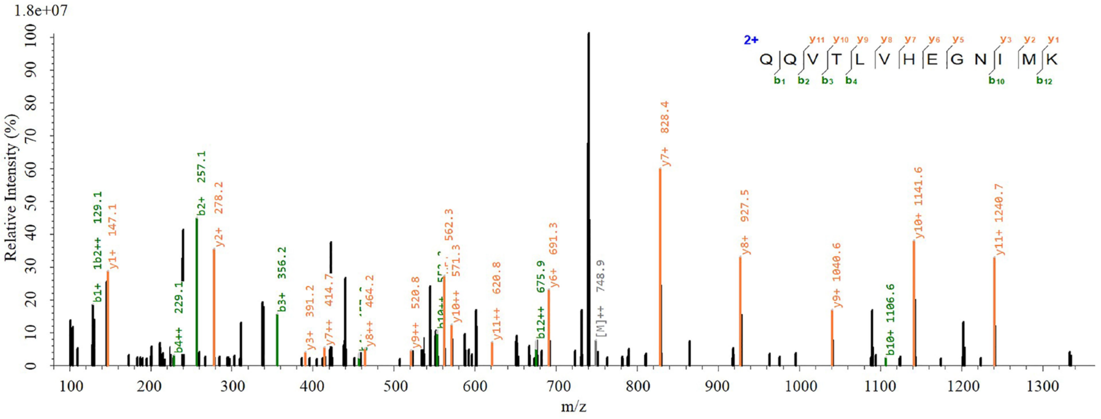

1.8e+07
Relative Intensity (%)
m/z
2+
Q Q V T L V H E G N I M K

D

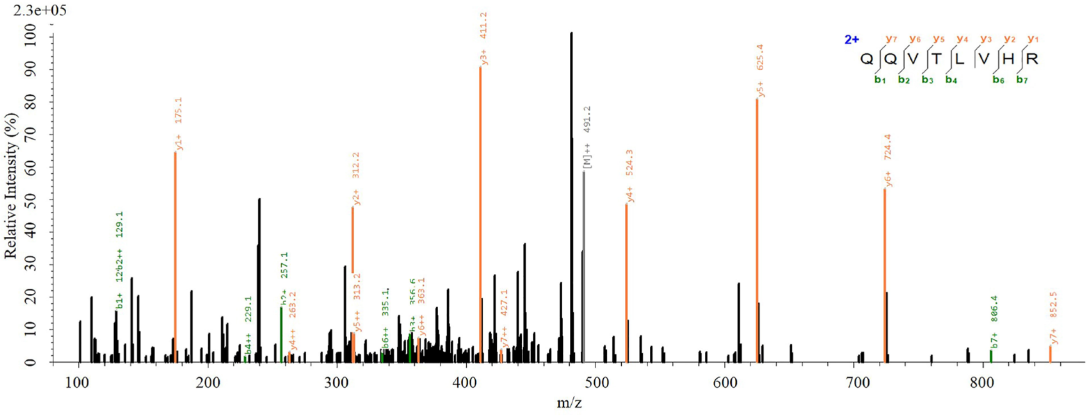

Figure S8. Mutations of IDH were verified by DNA sequencing and MS searches. (A) DNA sequencing verification site mutation. (B) K237 malonylation site in wild-type confirmed by MS analysis. (C) The K237E mutation site in the IDH-E mutant was confirmed by MS analysis. (D) The K237R mutation site in the IDH-R mutant was confirmed by MS analysis.

A

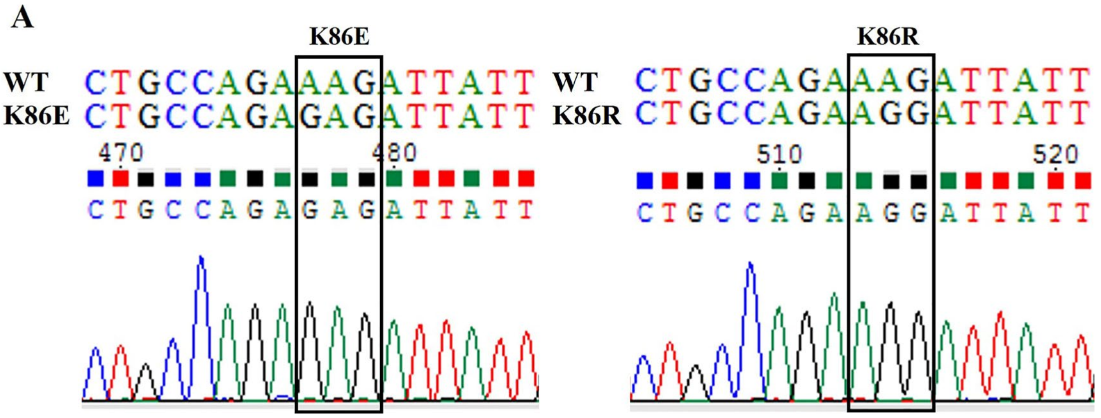

B

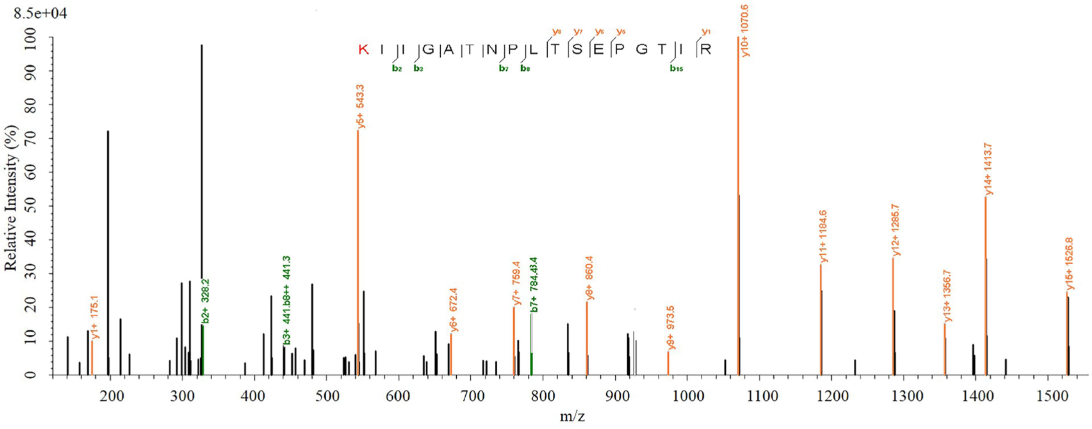

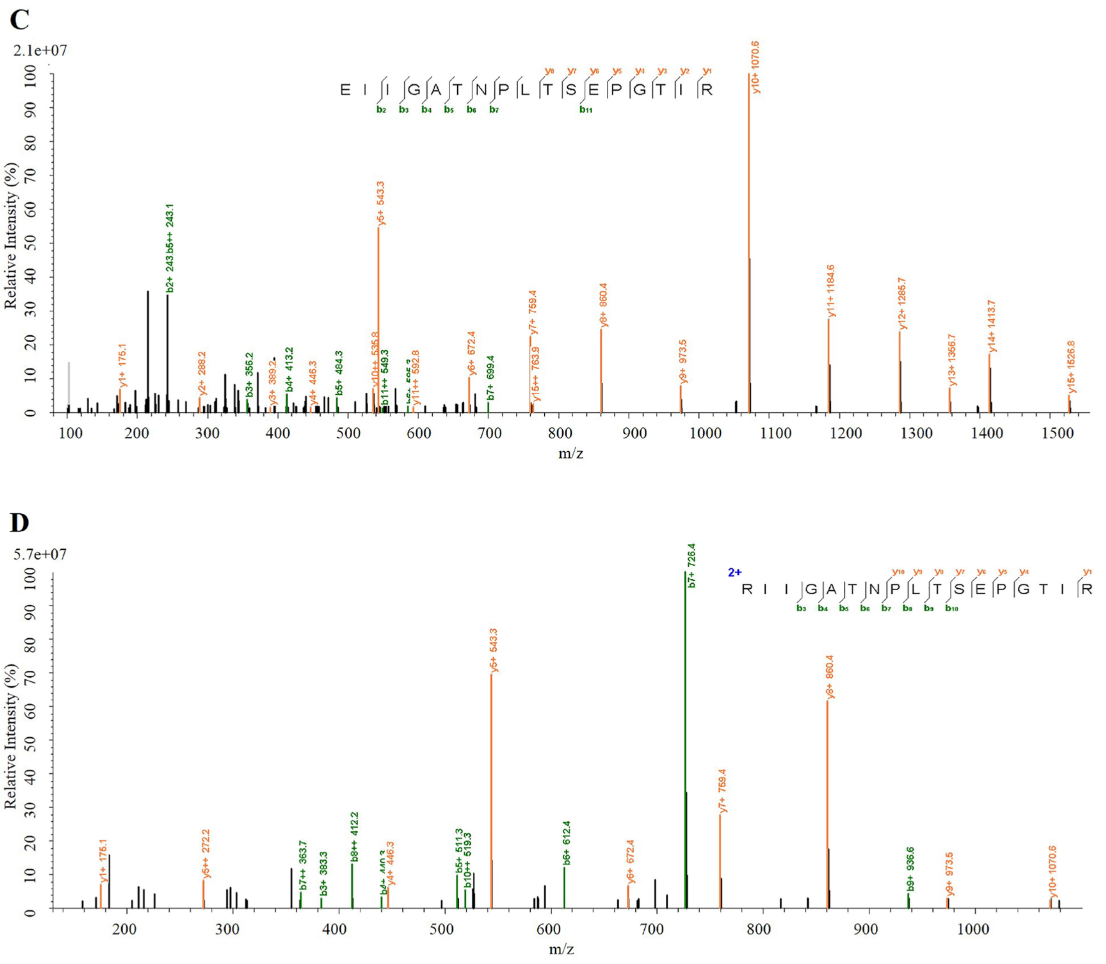

Figure S9. Mutations of NDK were verified by DNA sequencing and MS searches. (A) DNA sequencing verification site mutation. (B) K86 malonylation site in wild-type confirmed by MS analysis. (C) The K86E mutation site in the NDK-E mutant was confirmed by MS analysis. (D) The K86R mutation site in the NDK-R mutant was confirmed by MS analysis.

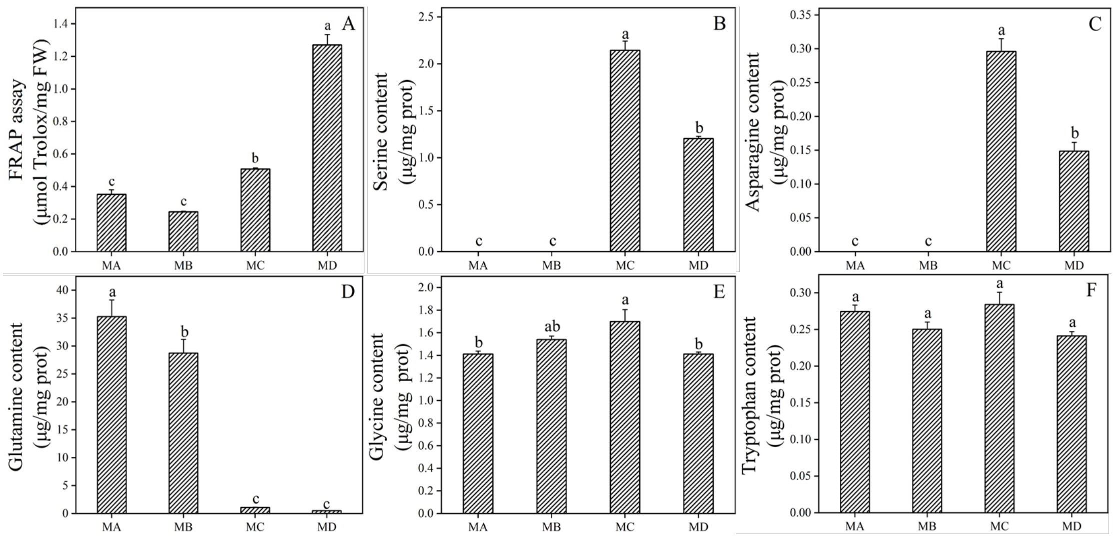

Figure S10. Changes of antioxidant capacity and amino acid content of *N. flagelliforme* exposed to different drought treatments. (A) ferric reducing antioxidant potential (FRAP); (B) serine, (C)

asparagine, (D) glutamine, (E) glycine, and (F) tryptophan content. The values are shown as mean ± standard error of three replicates and different letters represent significantly different values ($p < 0.05$).
